# Trend of RTS,S vaccine uptake in the malaria vaccine implementing programme (MVIP) pilot regions, Ghana; 2019–2022

**DOI:** 10.1016/j.heliyon.2024.e38858

**Published:** 2024-10-03

**Authors:** Michael Rockson Adjei, Peter Ofori Tweneboah, John Tanko Bawa, Janet Vanessa Baafi, Chrysantus Kubio, Kwame Amponsa-Achiano, Franklin Asiedu-Bekoe, Patrick Kuma-Aboagye, Martin Peter Grobusch, Sally-Ann Ohene

**Affiliations:** a Center of Tropical Medicine and Travel Medicine, Department of Infectious Diseases, Amsterdam University Medical Centers, Location AMC, University of Amsterdam, Amsterdam, the Netherlands; b World Health Organization, Country Office, Accra, Ghana; c PATH, Accra, Ghana; d Ghana Health Service, District Health Directorate, Sunyani West, Odumase, Ghana; e Ghana Health Service, Headquarters, Accra, Ghana; f Ghana Health Service, Regional Health Directorate, Volta Region, Ho, Ghana; g Institute of Tropical Medicine, and German Center of Infectious Diseases (DZIF), University of Tuebingen, Tuebingen, Germany; h Institute of Infectious Diseases and Molecular Medicine, University of Cape Town, Cape Town, South Africa; i Centre de Recherches Médicales en Lambaréné (CERMEL), Lambaréné, Gabon; j Masanga Medical Research Unit, Masanga, Sierra Leone

**Keywords:** District Health Information Management System, Ghana, malaria vaccine, malaria vaccine implementation programme, pilot study, RTS,S, vaccination coverage

## Abstract

**Introduction:**

The uptake trend of a new vaccine is unpredictable and may reflect the quality of introduction process and community acceptance. The objective of this study was to conduct a trend analysis of RTS,S malaria vaccine uptake in the seven pilot regions of Ghana from 2019 to 2022. The findings are envisaged to strengthen malaria vaccine introductions in the future.

**Methods:**

A retrospective analysis was conducted on routine childhood immunisation data for 2019–2022. Coverages for the first (RTS,S1), second (RTS,S2), third (RTS,S3) and fourth (RTS,S4) doses of malaria vaccine; third dose of diphtheria, tetanus, pertussis-containing vaccine (DTP3/Penta3); first dose measles-rubella (MR1) and second dose measles-rubella (MR2) vaccines were calculated. Dropout rates and uptake gaps were estimated to assess variations in the uptake of consecutive RTS,S schedules; and the differences in the uptake of RTS,S and the comparator vaccines, respectively.

**Results:**

Nationally, the coverages of the first three doses of the RTS,S malaria vaccine rose sharply from 2019 (RTS,S1 = 54.9 %; RTS,S2 = 54.6 %; RTS,S3 = 38.6 %) through 2020 (RTS,S1 = 70.7 %; RTS,S2 = 67.4 %; RTS,S3 = 66.3 %) to peaks in 2021 (RTS,S1 = 76.0 %; RTS,S2 = 73.1 %; RTS,S3 = 74.2 %), and declined marginally in 2022 (RTS,S1 = 74.0 %; RTS,S2 = 69.9 %; RTS,S3 = 71.3 %). For the fourth dose, the low uptake in 2020 (7.5 %) was followed by a steep rise in 2021 (46.9 %) that continued, but at a reduced rate to 50.6% in 2022. The dropout rates and uptake gaps were initially high but declined consistently over the study period. Generally, the trends in vaccination coverages, and dropout rates and uptake gaps at the national level were reflected in the respective regions.

**Conclusion:**

The coverage of RTS,S malaria vaccine improved consistently over the study period despite the low uptake in the early phase of the pilot. While the decreasing dropout rates and uptake gaps may indicate improved community acceptance, strengthening immunisation service delivery is crucial in sustaining the observed trajectory.

## 1 Introduction

Malaria is a leading cause of morbidity and mortality among young children and pregnant women and contributes significantly to disability-adjusted life years (DALYs) lost especially in resource poor countries [1,2]. Globally, malaria cases increased from 244 million in 2021 to 249 million in 2022 [3]. The World Health Organization (WHO) African Region accounted for approximately 95 % of the global cases with four countries – Nigeria, Democratic Republic of Congo, Uganda, and Mozambique contributing about 50 % of the burden [3].

Although malaria deaths have decreased steadily from 864,000 in 2000 to 608,000 in 2022, the percentage of total malaria deaths among children under five years has stalled since 2015, having declined from 87 % in 2000 to 76 % in 2015. Since then and as of 2023, there had been no change [3]. Approximately 48 % of the children under five years are exposed to malaria in Ghana [4], and 30 % of hospital admissions are attributable to the disease [5]. Approximately 20–30 % of the cases are severe [6].

Global commitment toward malaria elimination began with the launch of the Global Malaria Elimination Programme (GMEP) by the WHO in 1955 [7]. In May 2015, the Global Technical Strategy (GTS) was adopted by the World Health Assembly as a comprehensive framework to guide countries in their efforts to accelerate malaria elimination. The strategy sets the target of reducing global malaria incidence and mortality by at least 90 % by 2030 [8].

The GTS is confronted by the emergence of insecticide resistant mosquito species, and drug-resistant or at least tolerant *Plasmodium* species. The detection of *Anopheles stephensi* – an insecticide resistant species – in other parts of the world including Djibouti, Ethiopia, Sudan, Somalia, and Nigeria poses a great challenge to insecticide-based malaria control measures. Additionally, documentation of mutation-induced artemisinin partial resistance in the WHO African Region is of concern [3]. While novel therapeutic approaches are being explored to combat antimalarial resistance, there is optimism that the malaria vaccine would contribute significantly to mitigating the emergence of drug-resistant *Plasmodium* species [9,10].

**Fig. 1:**
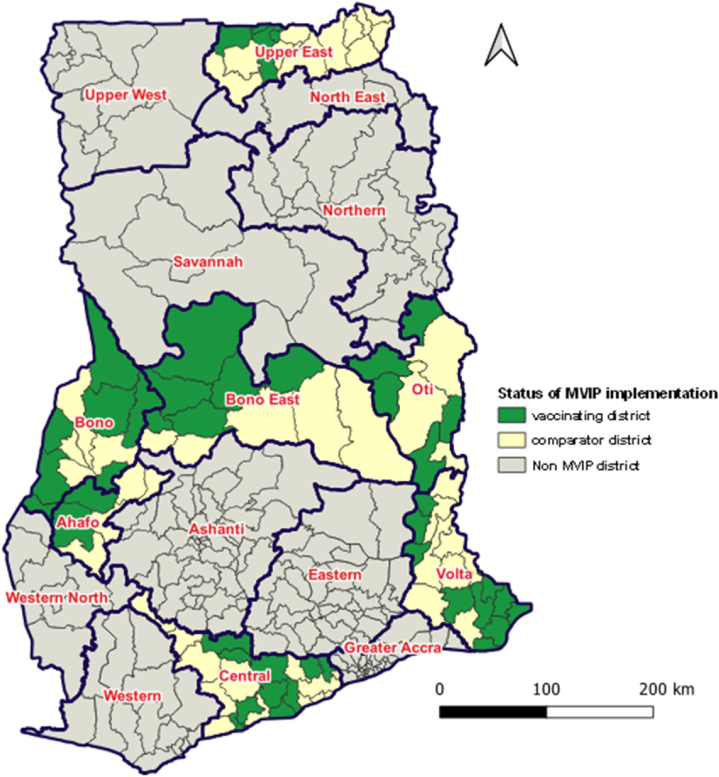
Malaria Vaccine Implementing Programme (MVIP) Pilot areas, Phase-1, Ghana; 2019–2022. Source: Adjei et al, 2021

Ghana along with Malawi and Kenya received WHO approval to pilot the introduction of the RTS,S malaria vaccine in 2019, to evaluate its feasibility, safety, and effectiveness before recommendation for wider use [11]. In Ghana, the vaccine was piloted in 42 districts of seven regions (Fig. 1) and was administered to children under five years in a four-dose schedule given at 6, 7, 9, and 24 months [12].

Many countries in the African Region are set to introduce the malaria vaccine into childhood immunization following WHO recommendation on the use of RTS,S malaria vaccine for prevention of *Plasmodium falciparum* malaria in children living in endemic areas with moderate to high transmission [13]. The uptake trend of a new vaccine is unpredictable and reflects the quality of introduction process and community acceptance [14]. Studies on the uptake trend of the malaria vaccine are limited, despite the potential of being a vital resource to new areas planning introduction. This study analysed the trend of RTS,S malaria vaccine uptake in the seven pilot regions of Ghana from 2019 to 2022. The findings are envisaged to strengthen malaria vaccine introductions in the future.

## 2 Methods

### 2.1 Study design

A retrospective analysis was conducted on routine childhood immunisation data for 2019–2022.

### 2.2 Health profile of study area

Ghana is a low-middle income country in sub-Saharan Africa with 16 administrative regions (Fig. 1). The 2021 population and housing census estimated the population as 30.8 million at a growth rate of 2.1 %, with children under five years constituting approximately 20 % (6,160,000) [15].

The country's immunisation programme – the Expanded Programme on Immunisation (EPI) – started in 1978 after the global programme was launched by the WHO in 1974 [16]. The EPI aims to protect all vulnerable populations against vaccine preventable diseases (VPDs) with particular focus on children and pregnant women living in Ghana. The EPI programme started with the introduction of vaccines to combat the so-called ‘six childhood killer diseases’ comprising measles, tuberculosis, diphtheria, pertussis, poliomyelitis, and tetanus [[16], [17], [18]]. Since then, the vaccine portfolio has expanded to 11 vaccines (including the RTS,S malaria and COVID-19 vaccines) protecting against diseases caused by 14 pathogens [16]. The EPI has made profound impact on child survival, having contributed to the significant reduction of under-5 mortality from 155/1000 in 1988 to 40/1000 in 2022 [18].

The RTS,S malaria vaccine was introduced in 42 out of 93 districts in Bono, Bono East, Ahafo, Central, Volta, Oti, and Upper East regions (Fig. 1). The selection of the implementing areas was based on a set of criteria recommended by the Ghana Malaria Vaccine Technical Working Group. These included: high malaria burden (*Plasmodium falciparum* prevalence rate, PfPr ≥20 %); strong EPI performance, malaria ecological zone, rural/urban mix; and adequate number of age-eligible children or target population to receive the vaccine [12]. The remaining (51) districts in the pilot regions served as comparator sites to facilitate evaluation of the malaria vaccine implementation programme (MVIP) objectives [12].

### 2.3 Data collection

The study data was extracted from the District Health Information Management System (DHIMS-2). DHIMS is a free open-source software (https://www.dhis2.org) developed from the District Health Information System (DHIS). It was first introduced in Ghana in the year 2007 and was primarily used for collecting and reporting health data. Data is collected at the health facility levels using standard registers, collated onto summary forms, and entered into the DHIMS platform weekly or monthly. The interface is password protected and accessible only by authorized healthcare managers.

**Fig. 2:**
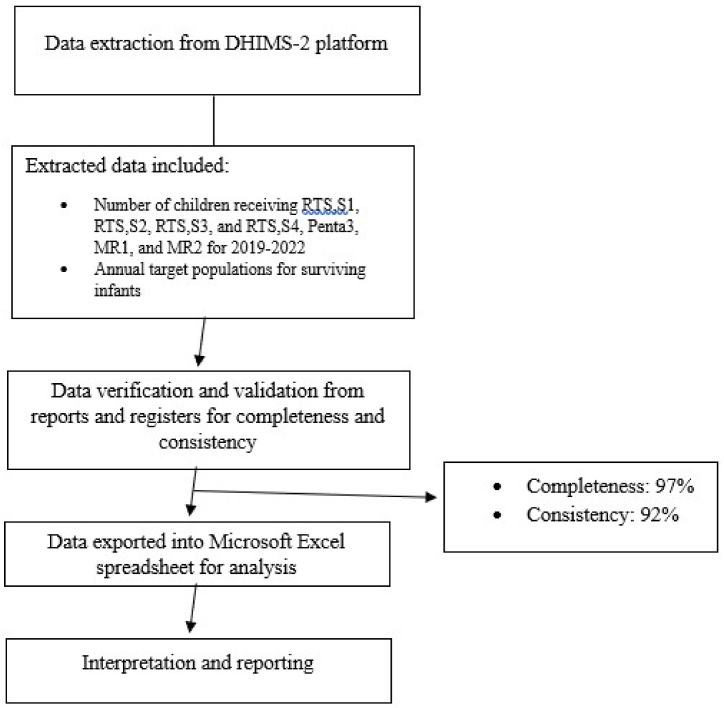
Study data management flow diagram, trend of RTS,S vaccine uptake, Ghana; 2019–2022.

The number of children vaccinated for the respective RTS,S vaccine schedules (first dose – RTS,S1; second dose – RTS,S2; third dose– RTS,S3; and fourth dose – RTS,S4); third dose of diphtheria, tetanus, pertussis (DTP)-containing vaccine (Penta3); measles-rubella (MR) vaccine (first dose – MR1; and second dose – MR2); and the annual target populations were extracted from the DHIMS-2 platform into Microsoft Excel 16.0 spreadsheet (Fig. 2). Data was verified from the source and validated, checking reports for completeness and consistency.

### 2.4 Data analysis

Data was analysed in Microsoft Excel 16.0 spreadsheet. Coverages for RTS,S1; RTS,S2; RTS,S3; RTS,S4; Penta3; MR1; and MR2 were calculated using the following expression: $\frac{Number\;of\;doses\;administered}{Target\;population}\;X\;100$.

The dropout rates and uptake gaps were estimated respectively, by the formula: $\frac{Number\;of\;doses\;administered\;for\;target\;vaccine-Number\;of\;doses\;adminsitered\;for\;comparator\;vaccine}{Number\;of\;doses\;administered\;for\;target\;vaccine}\;X\;100$ For example, dropout rate for RTS,S1/RTS,S3 was calculated by dividing the difference between doses administered for RTS,S1 and RTS,S3 with RTS,S1 multiplied by 100 [10,14]. Uptake gaps for RTS,S1/Penta3; RTS,S3/MR1, and RTS,S4/MR2 were calculated by similar approach.

The computations were done for each year of the study period (2019–2022). Results were displayed by place (region and national) and time using text, and charts.

## 3 Results

The average completeness and consistency of the study data with the primary sources were 97 % and 92 % respectively. A total of 1,471,895 doses of RTS,S malaria vaccine were administered nationwide over the following study periods – 11.3 % (165,690/1,471,895) in 2019; 24.7 % (363,097/1,471,895) in 2020; 32.3 % (475,391/1,471,895) in 2021; and 31.8 % (467,717/1,471,895) in 2022. Approximately 30.3 % (446,579/1,471,895), 29.1 % (428,113/1,471,895), 28.0 % (412,788/1,471,895), and 12.5 % (184,415/1,471,895) were administered for RTS,S1, RTS,S2, RTS,S3, and RTS,S4, respectively. The highest proportion (424,493/1,471,895; 28.8 %) of doses was administered in Central Region and the lowest (57,810/1,471,895; 3.9 %) in Upper East Region.

**Fig. 3:**
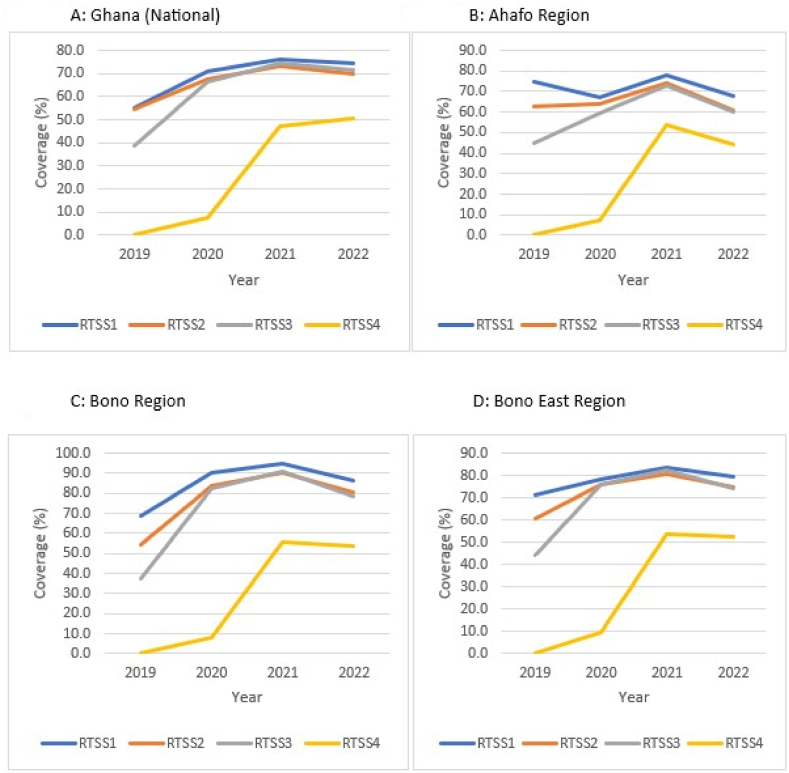

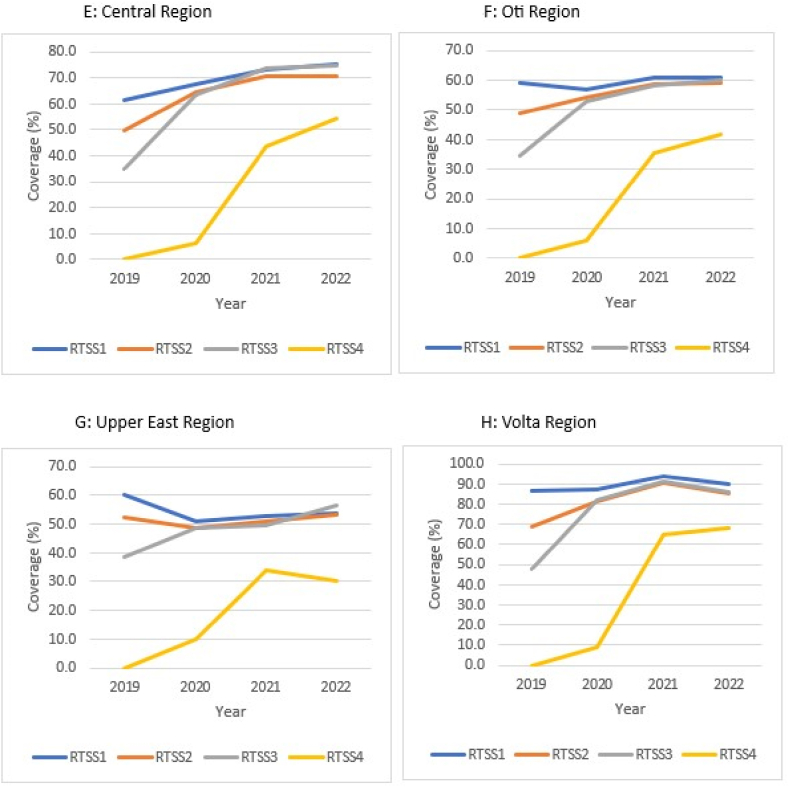
A–H: Trend of RTS,S malaria vaccination by region, Ghana; 2019–2022.

At the national level, the coverages of RTS,S1, RTS,S2, and RTS,S3 followed similar trends over the study period, rising sharply from 2019 (RTS,S1 = 54.9 %; RTS,S2 = 54.6 %; RTS,S3 = 38.6 %) through 2020 (RTS,S1 = 70.7 %; RTS,S2 = 67.4 %; RTS,S3 = 66.3 %) to peaks in 2021 (RTS,S1 = 76.0 %; RTS,S2 = 73.1 %; RTS,S3 = 74.2 %), and declined marginally in 2022 (RTS,S1 = 74.0 %; RTS,S2 = 69.9 %; RTS,S3 = 71.3 %). For the fourth dose, the low uptake in 2020 (7.5 %) was followed by a steep rise in 2021 (46.9 %) that continued, but at a reduced rate to 50.6% in 2022 (50.6 %) (Fig. 3A).

The coverage trajectory for the respective regions mimicked the pattern at the national level with sporadic variations. Slight dips in coverages occurred in 2020 for RTS,S1 in the Ahafo, Upper East, and Volta regions, respectively (Fig. 3B, G, and 3H). In 2022, the Central Region observed marginal increases in the RTS,S1 and RTS,S3 coverages, respectively (Fig. 3E); Volta Region witnessed some increases in RTS,S2 and RTS,S3 coverages, respectively (Fig. 3H); while Upper East Region recorded increases in all the three doses in the series (Fig. 3G). In the same year (2022), decline in RTS,S4 coverages were observed for Ahafo, Bono, Bono East, and Upper East regions (Fig. 3B, C, 3D, and 3G).

**Fig. 4:**
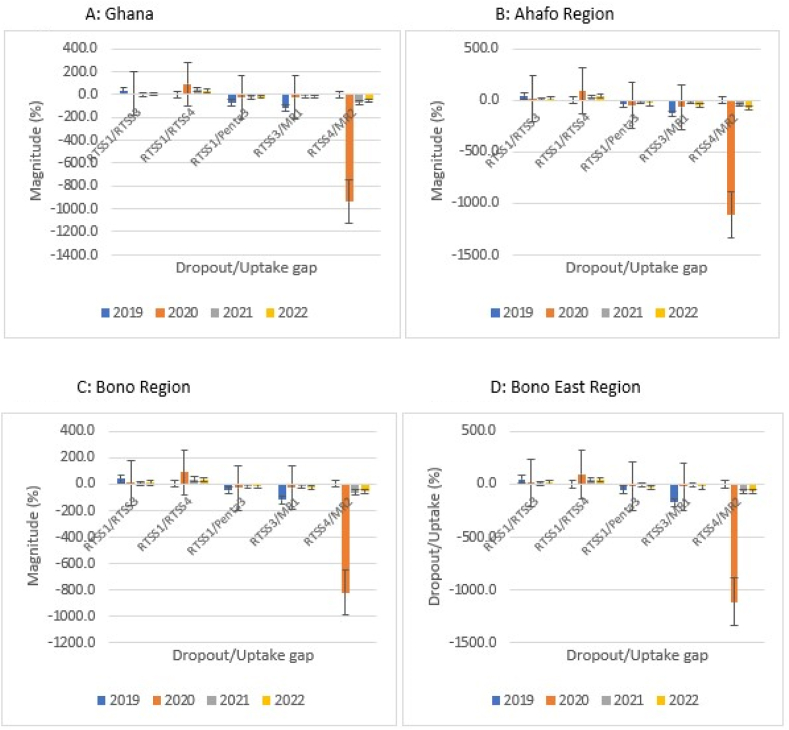

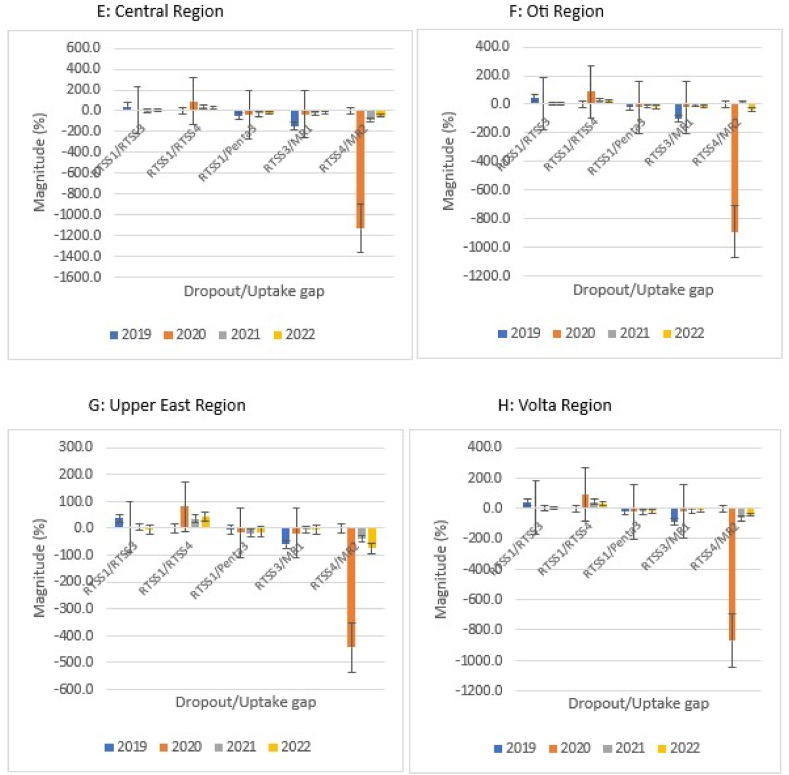
A–H. RTS,S malaria vaccine uptake gap and dropout rate by region, Ghana; 2019–2022.

Nationally, RTS,S1/RTS,S3 dropout rate decreased from 29.8 % (18,282/61,427) in 2019 to 2.4 % (3176/133,742) in 2021 and rose to 3.7 % (4858/130,247) in 2022. The dropout rate for RTS,S1/RTS,S4 rose to a peak of 89.4 % (108,332/121,163) in 2020 and declined thereafter to 31.6 % (41,176/130,247) in 2022. The dropout rates were higher for RTS,S1/RTS,S4 compared with RTS,S1/RTS,S3 over the study period. The uptake gaps increased with sequential doses of RTS,S in relation to the respective comparator vaccines. Wider gaps were observed for RTS,S4/MR2, RTS,S3/MR1, and RTS,S1/Penta3, respectively, in a decreasing order of extent. However, within each block (RTS,S1/Penta3, RTS,S3/MR1, and RTS,S4/MR2) uptake gaps declined consistently over the study period (Fig. 4A).

Generally, the dropout rates for the respective regions mirrored the national pattern (Fig. 4B–H). The magnitude of uptake gap between sequential RTS,S schedules in relation with comparator vaccines also followed the national pattern. Within the blocks, only Central and Volta regions, respectively, reflected the national pattern regarding variation in uptake gap over the study period (Fig. 4E and H).

## 4 Discussion

The present study sought to assess the uptake of RTS,S malaria vaccine over a 43-month period since its introduction in May 2019 in Ghana. The uptake of the malaria vaccine was low in the initial stages but improved consistently over the study period. It is worthy to note that Ghana's RTS,S malaria vaccine uptake trajectory patterned those of the other pilot countries, although there were slight differences in the coverages of the respective doses. We observed that, approximately one year after introduction of the vaccine in Ghana, nearly 70 % of the eligible children had received the first dose compared with 76 % in Malawi, and 82 % in Kenya, respectively. Similarly, 67 % had received the third dose compared with 64 % and 69 % in Malawi and Kenya, respectively [19,20]. The slight variations might be attributed to the differences in programme management, linked to factors including number of vaccinating areas and size of the target population, given that it is more challenging achieving higher coverages where the target population is huge or spread over a wider geographic area [20].

The uptake trend reflects the challenges and successes experienced with the malaria vaccine pilot in Ghana [[21], [22], [23]]. Ghana's immunisation programme, since its inception in 1974, had enjoyed public goodwill and vaccine hesitancy was a sporadic occurrence [11]. However, the malaria vaccine introduction faced hesitancy of disturbing magnitude, especially in the initial phase [11]. This was contrary to the findings of formative studies which indicated high caregiver support for the vaccine, with burden of malaria deaths being the key driver [24]. The low vaccine uptake was fueled by videos and messages circulated on social media portraying the vaccine to be unsafe, despite the approval by WHO and the national regulatory authority, Food and Drugs Authority [22].

Our study found high dropout rates and uptake gaps between successive RTS,S vaccine schedules, and between RTS,S and comparator vaccines, although these improved over time. While dropout rate may indicate poor continuity of service, uptake gaps indicate selective uptake of RTS,S vaccine. Thus some children received the comparators vaccines but not the RTS,S malaria vaccine, even though they were eligible. Apart from RTS,S3, the other doses in the series fell outside the regular childhood immunisation schedule (although Vitamin A is administered at age six months) and caregivers needed to make additional visits if their children were to complete the vaccination on time.

While the wide RTS,S1/Penta3 and RTS,S4/MR2 gaps may be attributed to the variable schedules of these vaccines, the same cannot be argued for the RTS,S3/MR1 gap since both (RTS,S 3 and MR1) are administered concurrently at nine months. Healthcare worker attitude and communication might account for the suboptimal uptake of the malaria vaccine. A study conducted by Yeboah et al. (2022) in Kasena Nankana Municipality in Upper East Region of Ghana found that caregivers who were not pleased with healthcare worker attitude were less likely to accept the RTS,S malaria vaccine although they accepted other vaccines [21]. The MVIP post-introduction evaluation also found that some healthcare workers specifically asked whether caregivers agreed to the administration of the malaria vaccine despite the same not being asked of the other vaccines [11]. Caregivers trust healthcare workers and whenever communication is shrouded in uncertainty, refusal is often a default response [21,25].

Other factors contributed to the observed dropout rates and uptake gaps. Fear of adverse events following immunisation (AEFI) was found as a key reason for refusal or lack of uptake continuity [26]. Again, the recommendation to continue with the use of other malaria interventions such as sleeping under insecticide-treated bed net every night was interpreted variedly by community members. . To some caregivers it meant the vaccine was not effective, hence the need for backup measure(s) [21].

Stockout of vaccines as observed in 2022, and inadequate operational funding affected reliability of service delivery and resulted in low uptake of the malaria vaccine [27]. These might have led to missed opportunities as caregivers received only a part of the service package and were unwilling to re-visit health facilities to complete the vaccination schedule [28]. Linked to the above observation was the phased introduction approach that made it challenging for caregivers to continue the RTS,S vaccination schedule after relocating from the vaccinating areas to communities that were not part of the pilot [22].

Barring the slight differences in the uptake trends between regions, the patterns largely reflected the national picture. A prime reason for the observation might be due to the harmonization vaccine introduction processes across the pilot areas. The national immunisation programme developed standard operating procedures (SOPs) and guidelines that were disseminated to the subnational levels to ensure operational uniformity [11].

The observed differences in the vaccination uptake patterns might be attributed to variations in implementation, given that concession was made for adoption of the SOPs and guidelines to context. Jingles were translated into other local languages by some of the implementing area, but this was not without the potential risk of message distortion. Again, the factors associated with RTS,S malaria vaccine uptake, though national, played out differently at the respective implementing areas. For example, the assertion that the vaccine was a birth control device had a significant negative effect on uptake in the regions where this misinformation and disinformation began [22]. Additionally, vaccine stock management issues at the subnational levels created artificial shortages and resulted in missed opportunities for vaccination.

The following were the limitations of the study: (1) routine data is often incomplete due to data management challenges including missing reports, although vaccination data is generally more accurate and complete compared to other indicators. However, given the high data completeness and consistency with the primary sources, it is unlikely that the data quality issues will significantly affect the study findings. (2) the vaccination uptake may not represent actual immunisation rates in the implementing areas due to contamination from other areas and/or administration of the vaccine to children outside the target age group. On the other hand, considering the negative uptake gaps, it is less likely that contamination had a weighty effect on the reported RTS,S coverages. (3) the low reliability of the population denominators might have affected the accuracy of the coverages. However, the inclusion of absolute number of doses administered (along with the coverages) is expected to clarify interpretation of the findings.

## 5 Conclusion

The study assessed uptake of the RTS,S malaria vaccine over a 43-month period in the pilot areas in Ghana. For the RTS,S malaria vaccine, the uptake was impressive given that coverages of newly introduced vaccines are often low in initial phases, mainly because of acceptability issues [14]. However, the dropout rates and uptake gaps decreased over the study period – an indication of improved community acceptance of the malaria vaccine. Factors including healthcare worker knowledge gap, attitude and communication towards caregivers, vaccine stock availability, caregiver perception on the safety of the vaccine, among others have been found to be associated with low uptake of malaria vaccine relative to the comparator vaccines [12,21].

The national immunisation programme should support the subnational levels to increase demand generation to make the malaria vaccine acceptable, as it is with the other childhood vaccines. Healthcare worker capacity should be strengthened though continuous in-service training to facilitate communication with caregivers and to manage community concerns on vaccine safety and effectiveness. The subnational levels should strenghten implementation of periodic intensification of routine immunisation (PIRI) activities in priority communities to catch up on missed children and improve coverage. The national immunisation programme should support the subnational levels to enhance vaccine stock management to avert spurious shortages and to ensure equitable vaccine distribution.

## Ethics approval and consent to participate

Ethics approval was sought from the Ghana Health Service Ethics Review Committee (ID number: GHS-ERC 002/10/23). Administrative permission was obtained from the Ghana Health Service for use of institutional data. Personal identifiers were removed to ensure anonymity, privacy, and confidentiality. The extracted data were stored on a computer protected with a password and used specifically for the study.

## Availability of data and material

The data that support the findings of this study are available from the Ghana Health Service. Data are available from the authors upon reasonable request and with permission of the Director General of the Ghana Health Service.

## Funding

This study did not receive any grant from funding agencies in the public, commercial, or not-for-profit sectors.

## Disclaimer

MRA, POT, and SAO are affiliated to the World Health Organization. The authors alone are responsible for the views expressed in this publication, and they do not necessarily represent the views, decisions, or policies of the World Health Organization.

## CRediT authorship contribution statement

**Michael Rockson Adjei:** Writing – original draft, Methodology, Investigation, Formal analysis, Data curation, Conceptualization. **Peter Ofori Tweneboah:** Supervision, Methodology, Investigation. **John Tanko Bawa:** Validation, Supervision, Methodology, Investigation. **Janet Vanessa Baafi:** Writing – review & editing, Writing – original draft, Validation, Methodology, Conceptualization. **Chrysantus Kubio:** Writing – review & editing, Supervision, Investigation. **Kwame Amponsa-Achiano:** Writing – review & editing, Validation, Supervision, Methodology, Investigation. **Franklin Asiedu-Bekoe:** Writing – review & editing, Validation, Supervision, Methodology, Investigation. **Patrick Kuma-Aboagye:** Writing – review & editing, Validation, Supervision, Methodology, Investigation. **Martin Peter Grobusch:** Writing – review & editing, Supervision, Methodology, Formal analysis, Conceptualization. **Sally-Ann Ohene:** Writing – review & editing, Supervision, Methodology, Formal analysis, Conceptualization.

## Declaration of competing interest

The authors declare that they have no known competing financial interests or personal relationships that could have appeared to influence the work reported in this paper.
